# Quantification of 15 Antibiotics Widely Used in the Critical Care Unit with a LC-MS/MS System: An Easy Method to Perform a Daily Therapeutic Drug Monitoring

**DOI:** 10.3390/ph14121214

**Published:** 2021-11-24

**Authors:** Catherine Feliu, Celine Konecki, Tristan Candau, Damien Vautier, Cyril Haudecoeur, Claire Gozalo, Yoann Cazaubon, Zoubir Djerada

**Affiliations:** 1 Department of Pharmacology, HERVI, EA 3801, SFR CAP-Santé, Reims University Hospital, 51 Rue Cognacq-Jay, CEDEX, 51095 Reims, France; catherine.feliu@univ-reims.fr (C.F.); ckonecki@chu-reims.fr (C.K.); tcandau@chu-reims.fr (T.C.); dvautier@chu-reims.fr (D.V.); chaudecoeur@chu-reims.fr (C.H.); cgozalo@chu-reims.fr (C.G.); 2 Institute Desbrest of Epidemiology and Public Health, INSERM, University Montpellier, 34000 Montpellier, France; yoann.cazaubon@chu-montpellier.fr; 3 Department of Pharmacology, Montpellier University Hospital, Avenue du Doyen Gaston Giraud, 34090 Montpellier, France

**Keywords:** therapeutic drug monitoring, mass spectrometry, antibiotics, multiparametric analysis

## Abstract

Potential under- or overdose of antibiotics may occur in intensive care units due to high variability in plasma concentrations. The risk is either treatment failure or toxicity. Thus, therapeutic drug monitoring of antibiotics may guide dosing adjustment, maximising antibacterial efficacy and minimising toxicity. The aim of this study was to develop and validate a method for the analysis of 15 antibiotics including beta-lactams, linezolid, fluoroquinolones, daptomycin, and clindamycin to have a complete panel in the management of infections. We proposed to develop a fast, sensitive, and quantitative method for the analysis of 15 antibiotics using ultra-performance liquid chromatography coupled with triple quadrupole mass spectrometer (UPLC-MS/MS) technology. this method required only 100 µL of plasma and consisted of a rapid liquid–liquid deproteinisation using methanol. Calibration curves ranged from 0.078 to 500 mg/L depending on the molecules, and were defined according to a therapeutic range. Inter- and intra-assay precisions values were less than 15%. This work described the development and the full validation of a precise, sensitive and accurate assay using UPLC-MS/MS technology. After validation, this new assay was successfully applied to routine therapeutic drug monitoring.

## 1. Introduction

Management of infections in intensive care unit patients is challenging and also associated with persistently poor clinical outcomes [1]. A recent study by Markwart et al. reviewed the burden of hospital-acquired sepsis, including in the critical care unit [2]. Their findings highlighted the important need for improved strategies in infection prevention and early diagnosis, and appropriate treatment to avoid progression to complications of sepsis. More severely, sepsis has been defined as a life-threatening organ dysfunction caused by a deregulated host response to infection [3]. Due to high mortality and morbidity, this topic is a global public health concern. The World Health Organisation has approved a global action plan to reduce antimicrobial resistance, including antibiotic resistance [4]. Among the strategies of the Action Plan on Antimicrobial Resistance, the optimisation of antibiotics use is a key focus of action [5].

There is high variability in the pharmacokinetic parameters of antibiotics in critical care patients [1,6]. Some pathophysiological conditions, such as inflammation associated with sepsis, shock, organ failure such as liver or kidney failure, can modify the pharmacokinetics of antibiotics in the critical care unit. Care protocols, such as vascular replacement, administration of catecholamines, mechanical ventilation, extracorporeal circulation, or extra-renal purification are also described as influencing the pharmacokinetic parameters of antibiotics [6,7]. Due to the unstable status of patients in critical care units, there is intra-individual variability in addition to inter-individual variability. The distribution and elimination of antibiotics in these patients are therefore unpredictable. This may result in highly variable plasma concentrations despite adherence to recommended dosing regimens. This can cause a potential under- or overdose of the drug, with either a risk of treatment failure or toxicity. The DALI (Defining Antibiotic Levels in Intensive care unit patients) study aimed to determine whether target concentrations of β-lactams in critically ill patients were achieved and to determine whether concentrations were associated with patient outcomes [6,8]. In this study, performed on eight beta-lactams used in the intensive care unit (ICU), the authors demonstrated that less than 50% of patients achieved the predefined PK/PD target [8]. They also demonstrated that patients with sub-therapeutic antibiotic plasma concentrations had a lower probability of a positive clinical outcome [8]. Regarding the management of antibiotics in the critical care unit, the probability of reaching the PK/PD target has been reported to be low in different clinical pathophysiological settings [9]. There is increasing evidence in the literature of the benefits associated with achieving PK/PD goals in terms of reducing mortality, clinical cure, reduced length of stay, and reduced toxicities [8,9,10,11]. Several PK/PD targets of antibiotics, including beta-lactam antibiotics [6,8,10,11], linezolid [12,13], daptomycin [14,15], and fluoroquinolones [16,17,18] have been studied in critically ill patients

Thus, the level of evidence for the therapeutic drug monitoring (TDM) of antibiotics is strong, with increasing interest [7,19]. TDM may guide dosing adjustment and aims at maximising antibacterial efficacy, demonstrating the impact on clinical outcomes, and minimising toxicity. Numerous studies have been published for the TDM of antibiotics, mainly about liquid chromatography. Many quantification methods using liquid chromatography coupled with UV detection were developed, but selectivity must be properly evaluated as interference may occur when patients are polymedicated [20]. Due to the improvement of technology, better selectivity, precision, and sensitivity were achieved with liquid chromatography coupled with mass spectrometry (LC-MS). LC-MS is now widely used for the TDM of immunosuppressants, but also in the field of pharmacology of anti-infective drugs (antifungals, antiretrovirals and antibiotics), as well as in neuropsychopharmacology [21,22,23,24,25]. Many analytical procedures were developed for the TDM of antibiotics. While some publications proposed the analysis of one class of antibiotics, such as beta-lactams [20,26], oxazolidinone [27,28], or daptomycin [29,30], others reported methods for the concomitant analysis of several classes of antibiotics [31,32,33,34,35,36,37]. Appendix 5 of the Wellington ICU Drug Manual depicted an overview of antibiotic sensitivity of different bacteria incriminated in the intensive care unit [38]. Different antibiotics were recommended for the management of bacterial infections in intensive care patients, depending on the bacteria involved. For example, clindamycin was indicated for the management of meticilline-sensitive or meticilline-resistant *Staphylococcus aureus* infections, but also *Streptococcus*, *Clostridium* and *Bacteroides* infections [38]. Thus, the aim of this study was to develop and validate an easy-to-implement sensitive and quantitative method for the analysis of 15 antibiotics to have a complete panel for the management of all these infections, including beta-lactams, linezolid, fluoroquinolones, daptomycin, and clindamycin. We selected antibiotics that are widely used in the critical care unit and for which the benefit of achieving PK/PD targets has been studied [8,9,10,11,13,15,16]. Ultra-performance liquid chromatography coupled with a tandem mass spectrometer (LC-MS/MS) was optimised and then validated to quantify amoxicillin, aztreonam, cefazolin, cefepime, cefotaxime, cefoxitin, ceftazidime, ciprofloxacin, clindamycin, daptomycin, ertapenem, linezolid, meropenem, ofloxacin, and piperacillin. 

## 2. Results and Discussion

### 2.1. Optimisation of the Method

First, the mass spectrometer was optimised. Preliminary experiments were performed to optimise the source parameters, such as auxiliary gas, sweep gas, flow rate, spray voltage, positive ion, ion transfer capillary temperature, and vaporiser temperature. After source optimisation, different parameters of the multiple reactions monitoring (MRM) mode of acquisition were optimised for each compound: RF-lens, energy of collision, and two MRM transitions were selected: the first for the quantification and the second for the confirmation of the analyte (Table 1).

After this step, different chromatographic parameters were tested and optimised to achieve a chromatography process that provided efficient elution of all compounds. A neat solution at the concentration of 100 mg/L, except for ciprofloxacin, clindamycin, linezolid, and ofloxacin (10 mg/L), was used to test two different Acquity UPLC^®^ columns (HSS T3 1.8 µm 2.1 mm × 50 mm and HSS T3 1.8 µm 2.1 mm× 150 mm Waters Corp., Milford, MA, USA). Acquity HSS T3 columns are compatible with 100% aqueous mobile phase. As previously described, among the chromatographic column, this column exhibits a strong analytical performance for the separation of polar and non-polar compounds [21,34,39]. Indeed, other columns (Acquity BEH HILIC 1.7 µm 2.1 mm × 50 mm and BEH C18 1.7 µm 2.1 mm × 50 mm, Waters Corp., Milford, MA, USA) were tested without improvement of the chromatographic separation (analytical run, shape of the chromatographic peak, data not shown). The column allowing the optimal chromatographic resolution was selected. The analysis time was longer in the 150 mm column without improving compound separation. Thus, the HSS T3 1.8 µm 2.1 × 50 mm column was chosen. The mobile phases used were water and 0.1% formic acid (phase A) and acetonitrile and 0.1% formic acid (phase B). Mobile phase tests were performed with and without ammonium formate or ammonium acetate. The benefit of these ammonium derivatives is to buffer the mobile phase for some compounds of interest that would be sensitive to pH variation. As described, ammonium adducts can also, in some applications, improve the sensitivity of the method [34]. Different concentrations of ammonium acetate and formate were tested. Two and 10 mM failed to show any improvement compared to a mobile phase without ammonium derivatives. Thus, the phases selected were water and 0.1% formic acid (phase A) and acetonitrile and 0.1% formic acid (phase B).

Different gradients were tested to optimise peak shape and resolution. With the first linear gradient tested (from 95/5 phase A/phase B to 5/95 between 0 and 4 min), the shape of the peaks for meropenem and ertapenem was not acceptable because of an undesired tailing. Despite this feature, elution and peak resolution were correct. Other tests were carried out by adjusting the phase percentages: 98/2 to 5/95 and then 100/0 to 5/95 from 0 to 4 min. Increasing water proportions at the beginning of the gradient significantly improved the shape of the penems peaks. Starting the gradient with 100% water was retained. Daptomycin was the last compound eluted at 3.52 min. The gradient was therefore adapted to obtain the same slope as in the tests, and then at 3.6 min, the gradient was switched to 95% acetonitrile for a 30 s rinse period. Finally, a re-equilibration period of 1.2 min at 100% water was programmed to match about 2.5 times the column’s dead volume. 

Several injections performed in full-scan acquisition mode allowed us to check that the flushing step was correct and efficient. Once the chromatography was optimised, some parameters of the mass spectrometer were optimised once again: the MRM windows were set to retention time ± 0.5 min, and finally, the dwell time was set to have at least 15 points per peak at standard 3.

**Figure 1:**
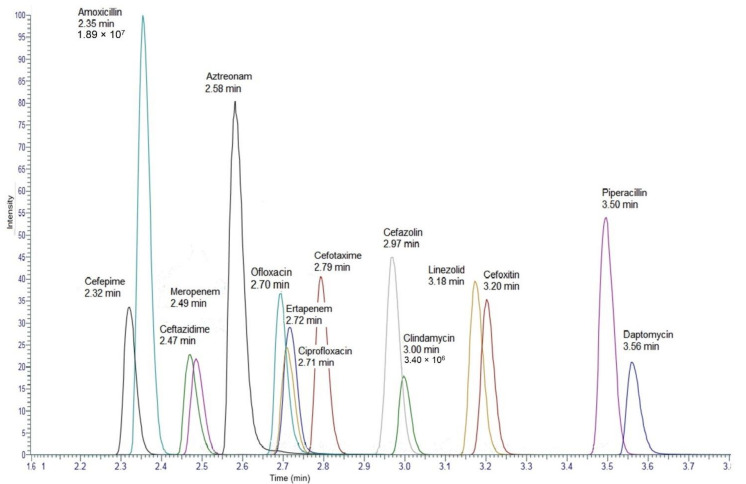
Reconstructed chromatograms for all analytes of the standard 6. Minimal signal intensity was observed for clindamycin (3.40 × 10^6^) and the maximum signal intensity was observed for amoxicillin (1.89 × 10^7^).

Thereafter, sample pre-treatment was optimised. Deproteinisation processes were assayed using 300 µL of methanol or 150 µL methanol/150 µL acetonitrile. Extraction recovery for all molecules was greater than 75% except for aztreonam (20%). Recovery was improved for ofloxacin, linezolid, meropenem and cefoxitin, with a signal increase between 5 and 20%, according to the compounds, with the methanol preparation. For the other molecules, the extraction recovery was equivalent between the two deproteinisation strategies. Pre-treatment with methanol was retained because it allowed obtaining satisfactory signals for all the compounds. Finally, different dilution tests and injection volumes were tested. The selected process was that 50 µL of supernatant was diluted in 200 μL of phase A and 2 µL was injected. Figure 1 depicted reconstructed chromatograms of the standard 6 for all analytes.

Amoxicillin-D_4_, cefazolin-^13^C_2_^15^N, meropenem-D_6_, ofloxacin-D_8_, ciprofloxacin-D_8_, piperacillin-D_5_, cefotaxime-D_3_, and linezolid-D_3_ were chosen as internal standards. The internal standards were selected to belong to different pharmacotherapeutic classes and to be distributed throughout the chromatogram. The selection of the different internal standards was justified by their ability to correct and reproduce the analytical behaviour of each antibiotic.

The several steps of optimisation allowed us to have a short run suitable for a daily TDM activities. We opted for this strategy despite an incomplete chromatographic resolution for some analytes. However, the mass spectrometer detector compensated without loss of sensitivity nor matrix effect. The number of points under the peak was greater than 20 for all analytes. All these choices have been validated by analytical performance results that fulfil all FDA (U.S. Food and Drug Administration) and EMA (European Medecines Agency) validation criteria [39,40].

### 2.2. Validation of the Method

#### 2.2.1. Linearity, Precision, and Accuracy

A study of the calibration regression model was performed. The model that provided the lowest bias on three levels of quality controls (Table S1), the best R^2^ and the lowest absolute sum of square (Table S2) was selected for each compound. The extra-sum-of-squares F test was also performed to select the simplest model and a *p* value less than 0.2. The residual distributions were depicted for both the linear and quadratic regression model (y_observed_ − y_theorical_ as function of different concentration levels) (Figure S1). The most appropriate regression model for all molecules was the quadratic model. This is particularly obvious in the residual distribution analysis. In most cases, the data showed systematic bias in the linear model. One explanation for this is that our MS/MS detector was very sensitive, with a rapid evolution of the detector’s response between very low and high concentrations. Another explanation may be that we aimed for a large calibration range from low to very high concentrations.

For all analytes, quadratic regression (Y = ax^2^ + bx + c) without weighting satisfied all predefined criteria [21,41] (Table 2, Supplementary File: Tables S1 and S2 and Figure S1). Over the considered concentration range, the regression coefficient (r^2^) of the calibration curves were always greater than 0.998 (*n* = 6, Table 2) with back-calculated calibration samples within ±15% (±20% at LLOQ) of nominal concentration. The precision and accuracy (*n* = 6) of the LLOQ and ULOQ for each analyte were within the recommended limits (Table 2). The relative standard deviations of quality controls (Table 3) were within 0.9–12.5% for both intra- and inter-assay precision (*n* = 10) and were within acceptance criteria [39,40]. Evaluation of quality control accuracy showed a relative standard deviation (*n* = 10) less than ±15% (85.9–114.4%) from the target concentration at each tested level (Table 3).

#### 2.2.2. Specificity and Selectivity

**Figure 2:**
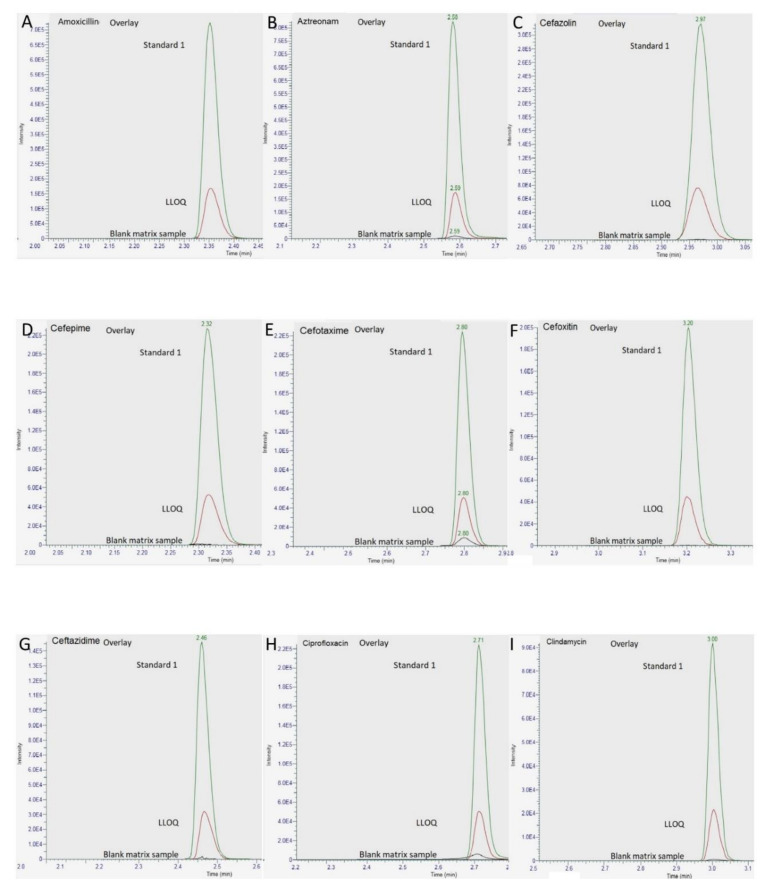

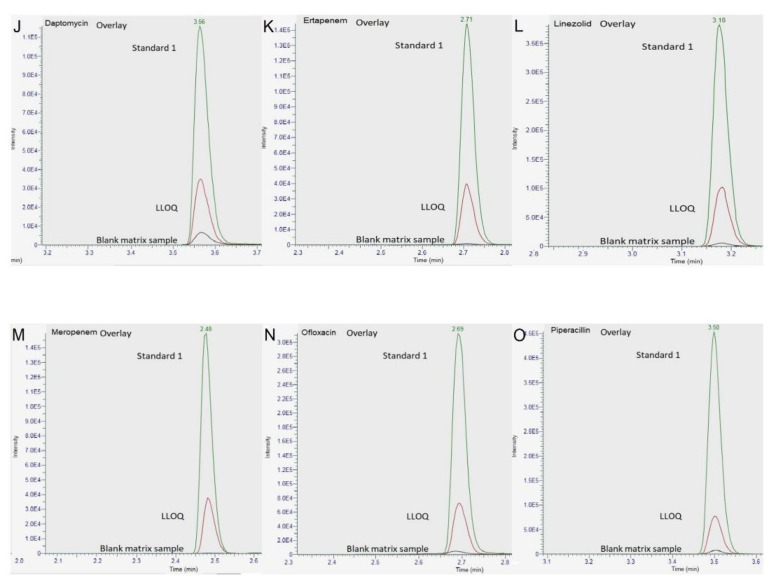
Overlapping of standard 1, LLOQ and blank-extracted chromatograms of amoxicillin (**A**), aztreonam (**B**), cefazolin (**C**), cefepime (**D**), cefotaxime (**E**), cefoxitin (**F**), ceftazidime (**G**), ciprofloxacin (**H**), clindamycin (**I**), daptomycin (**J**), ertapenem (**K**), linezolid (**L**), meropenem (**M**), ofloxacin (**N**) and piperacillin (**O**).

Analysis of six different blank plasma samples did not show any interference (<5% of LLOQ and IS response) at the retention time windows for each specified MRM. For each sample, the response was less than 20% of the LLOQ for analytes and 5% for internal standards. The overlap of standard 1, LLOQ, and blank-extracted chromatograms for all compounds are depicted in Figure 2. Focus on overlay (LLOQ and matrix blank) for ertapenem and clindamycin are depicted in the (supplementary file Figure S2). 

#### 2.2.3. Matrix Effect

**Figure 3:**
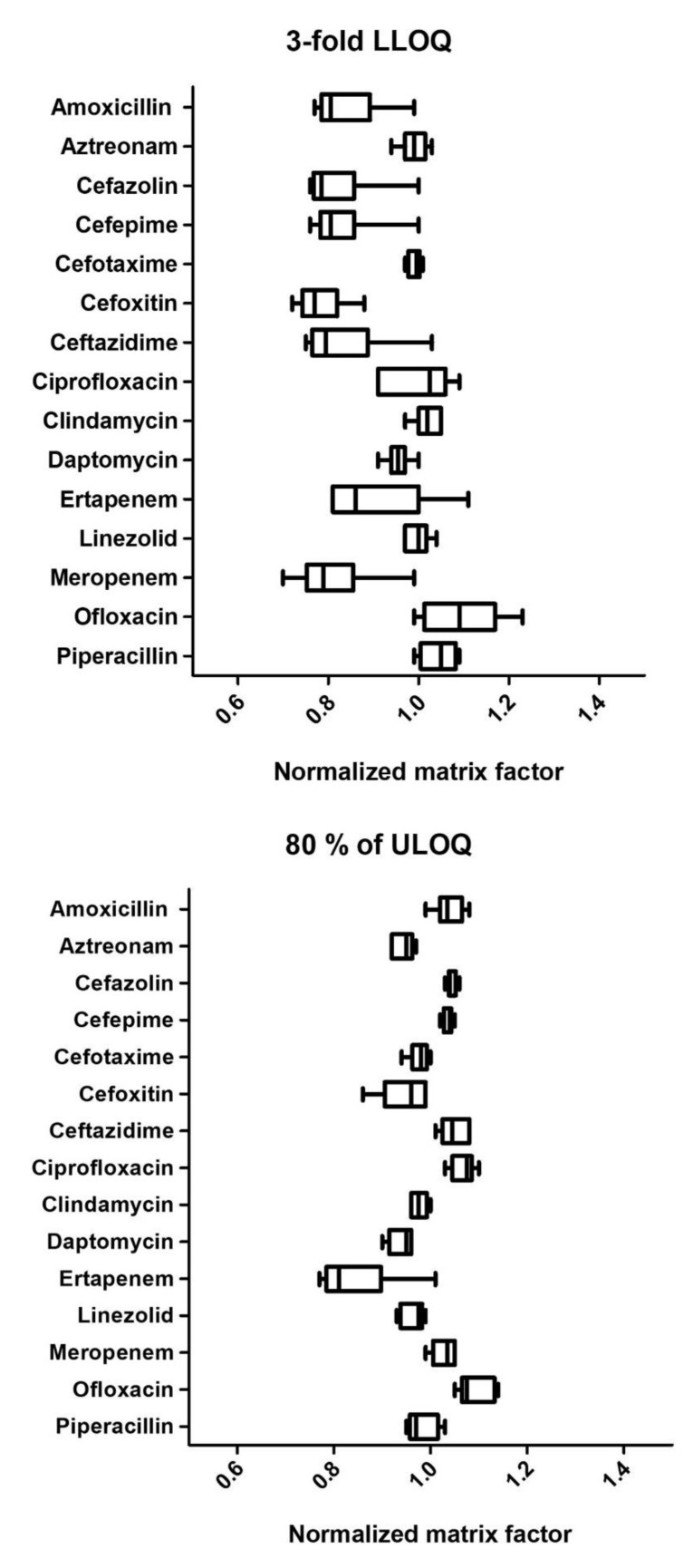
Matrix effect at low and high concentration levels (*n* = 6): 3-fold LLOQ and 80% ULOQ). Results are expressed as box and whiskers (min to max) (*n* = 6/group).

Matrix factor (MF) ranged from 0.72 to 1.14 and 0.83 to 0.98 at the concentration of 3-fold LLOQ and 80% of ULOQ, respectively (Table S3). For both concentrations, the relative standard deviation was less than 15% (0.6–14.8%). A matrix effect associated with a loss of signal was observed for amoxicillin (MF 0.72), cefazolin (MF 0.73), cefepime (MF 0.74), cefoxitin (MF 0.72), ceftazidime (MF 0.76) and ertapenem (MF 0.75), and meropenem (MF 0.72), only at the concentration of 3-fold LLOQ. The use of the deuterated internal standards was sufficient to correct the matrix effect, since IS normalised matrix factor ranged from 0.82 to 1.11. As the recommendation states, the relative standard deviation of the normalised matrix factor was less than 15% for each antibiotic (0.9–11.8%). Figure 3 depicted for each analyte the normalised matrix factor, described by the median and interquartiles. 

#### 2.2.4. Stability

Standard solutions and IS solutions were evaluated to be stable for 5 days at +4 °C For each compound, the mean and relative standard deviation (RSD, %) of areas under the curve of standard 6 over 5 days were displayed in Table S4 (*n* = 5). Assays of freeze-dried quality controls have shown stability for at least 3 months at −20 °C. Assays conducted on the reconstituted quality controls have demonstrated stability for at least seven days at −20 °C. Finally, post-preparative stability was evaluated by keeping standard calibrators in an auto sampler (+10 °C) for 3 h, 6 h, 12 h and 24 h. Comparison of the normalised areas to the internal standard provided 24 h post-analytical stability for all compounds except for ertapenem (3 h) (*n* = 6, Table S4).

#### 2.2.5. Carry-Over Effects

To assess contamination, residual peak area of a blank sample analysed after the highest standard was compared with the signal of the LLOQ (*n* = 6). For all the analytes, as recommended [39,40], the remaining area was less than 2%, thus less than the recommended level of 20% of the LLOQ and less than 5% of the signal of the internal standard. These results confirmed the absence of contamination and therefore adequate chromatographic conditions.

### 2.3. Applicability

This fast and accurate assay allowed us to propose a TDM in accordance with current recommendations [38]. For all bacterial infections in a patient in intensive care unit, we can offer a TDM of one or more antibiotics strategies.

#### 2.3.1. Example

This assay was successfully applied for TDM of antibiotics in plasma. A data collection analysis was carried out over 3 months for the antibiotics for which we had started the TDM proposal. TDM was performed on 137 patients who were admitted to a care unit of the University Hospital of Reims. Two hundred and seventeen quantifications of antibiotics in plasma were undertaken. The results and timing at which the samples were drawn were listed in Table 4. The concentrations were compared to reference values reported in the literature [7,31,33,36]. During this period, 27 measurements of cefepime were performed. In 48.2% of cases, the concentrations were within normal values (5–35 mg/L). In 40.7% of cases, the concentrations were higher than recommended (>35 mg/L) and in 11.1% of cases, lower (<5 mg/L). High concentrations were non-significantly associated with moderate or severe renal failure. In 28% of cases, neurological signs such as confusion, sedation, and hallucination were observed in patients. The quantification of cefotaxime and ceftazidime revealed, as for cefepime, approximately 40% of values above the recommended values, and thus an associated higher risk of neurotoxicity. Regarding amoxicillin, the majority (51.1%) of the quantifications performed showed concentrations below the recommended therapeutic values. Concerning carbapenems, even if we had little data, our results showed an overdosage of 100% for ertapenem and 33.3% for meropenem. All these results confirmed the need to carry out a TDM of antibiotics in order to promote their appropriate use as requested by international authorities and scientific societies [4,5,7].

For illustration, a 72-year-old male patient was admitted for the management of a postoperative *Staphylococcus aureus* and *Pseudomonas aeruginosa* infection on a total knee replacement. Antibiotic management was an association of doxycyclin (per os 100 mg twice a day), sulfamethoxazole 800 mg and trimethoprim 160 mg (per os three times a day) and ceftazidim (intravenous 2 g three times a day). Three days after the introduction of ceftazidime, behavioral disorders such as agitation and hallucinations were observed in this patient. As the accumulation of ceftazidime is known to be responsible for neurotoxicity [42], therapeutic drug monitoring was performed and identified an accumulation of ceftazidime with a plasma concentration of 196.8 mg/L (recommended targeted concentration 35–80 mg/L [7]). At the same time, renal function was impaired, with creatinine clearance decreasing from 82 mL/min to 45 mL/min in 10 days. Acute renal failure is one of the main risk factors of β-lactam accumulation with a risk of neurotoxicity [43].

Another example is a 78-year-old-female patient admitted to the intensive care unit for septic shock due to cholecystitis. *Escherichia coli* ESBL was identified, and antibiotic therapy with ertapenem of 1g per day was introduced. Due to instability of renal function and worsening to acute renal failure (glomerular filtration rate of 39 mL/min), therapeutic drug monitoring was performed. The plasma concentration of ertapenem was 11.17 mg/L after administration of the second dose (recommended targeted concentration 5–10 mg/L [7]). After two days of treatment, neurological deterioration with confusion was observed in the patient. Renal function was still deteriorating. Monitoring of concentration of ertapenem was performed and identified an accumulation of ertapenem with a concentration of 29.7 mg/L, which was three times greater than normal [7]. Therefore, the neurological deterioration was attributed to the accumulation of ertapenem in this patient. Ertapenem was interrupted for 1 day and then a dosage adjustment to 0.5 g per day was proposed. The ertapenem concentration was monitored again two days later at 8.05 mg/L.

These two examples illustrate the importance of therapeutic drug monitoring, particularly in patients with unstable pharmacokinetic parameters.

We present a third example illustrating the relevance of a multi-parametric analysis. An 83-year-old female patient was consulted after a cat bite to the left calf. Treatment with pristinamycin was initiated. Four days later, she was admitted to the hospital for an altered health status associated with asthenia, anorexia, nausea, and fever. The diagnosis was *Pasteurella multocida* bacteremia complicated by mitral valve endocarditis. The patient was treated with amoxicillin (12 g per day) and ofloxacin (200 mg twice daily) for 6 weeks. In the context of altered renal function in this patient (glomerular filtration rate of 59 mL/min), therapeutic drug monitoring was carried out. Firstly, amoxicillin was at a level of 65 mg/L (recommended targeted concentration 10–80 mg/L [7]) and ofloxacin was at 4.25 mg/L. The next day, the residual concentration of amoxicillin was increased to 114.5 mg/L and ofloxacin to 8.05 mg/L. A dosage adjustment by decreasing the doses of amoxicillin was proposed. The therapeutic drug monitoring performed after dosage adjustment allowed to quantify amoxicillin at 62.3 mg/ and ofloxacin at 7.62 mg/L.

#### 2.3.2. Comparison with Reported Methods

Using UPLC-MS/MS technology, our assay was developed and was validated to simultaneously quantify 15 antibiotics, including 9 beta-lactams, requiring only 100 μL of a plasma sample with a chromatographic run time of 5.50 min. The simultaneous determination of antibiotics was also recently described by using other procedures [33,34,35,37]. All these published assays have proposed a multi-parametric method to quantify antibiotics in plasma. Like us, the methods of Decosterd et al. [34] and Lefeuvre et al. [33] required 100 µL of plasma, while Barco et al. [37] and Ferrari et al. [35] needed only 50 µL. Decreasing the assay volume is a perspective of improvement of this work. This could be of great interest, especially in the pediatric population. Regarding sample preparation, except for Ferrari et al. who used a commercial MassTox^®^ TDM Series A basic kit (Chromsystems Instruments & ChemicalsGmbH; Gräfelfing, Germany), all other techniques performed a deproteinisation in methanol, sometimes followed, as we do, by dilution in water [33,34]. The dilution factors varied according to the techniques. The chromatographic conditions also varied. Lefeuvre et al. [33] and Barco et al. [37] performed their chromatographic separation with an Accucore C18 column (Thermo Fisher Scientific, San Jose, CA, USA), with [33] or without ammonium formate in the aqueous mobile phase [37]. Like us, Decosterd et al. [34] performed chromatographic separation using Acquity HSS T3 column (Waters Corp; Milford, MA, USA). However, the preparation of the aqueous mobile phase differed, with 10 mM ammonium formate in ultrapure water +0.4% formic [34], whereas in this present study, the mobile phase only consisted of water +0.1% formic acid (*v*/*v*). Regarding the mass spectrometer, all these techniques used the MRM mode of acquisition, except Lefeuvre et al. [33], who performed high-resolution mass spectrometry mode acquisition. With an acquisition time of 5.5 min, our method is the second fastest of the described techniques, with a minimum acquisition time of 5 min [37] and a maximum of 9 min [33.34]. Concerning the list of drugs proposed by each method, we also noted the specificities of each work. Ferrari et al. proposed the quantification of four compounds, including two beta-lactams, linezolid and teicoplanin. Decosterd et al. [34] proposed a method quantifying 12 molecules, including nine beta-lactams associated with rifampicin and daptomycin. Lefeuvre et al. [33] proposed a technique allowing the quantification of 11 beta-lactams associated with two fluoroquinolones and clindamycin. Barco et al. [37] proposed the most varied technique in terms of antibiotic classes. They proposed a technique allowing the simultaneous quantification of three aminoglycosides, two glycopeptides, linezolid, tigecycline, and ciprofloxacin but only four beta-lactams. Our method required only 100 µL of sample, and after a simple and fast pretreatment, offers quantification of 10 beta-lactams associated with two fluoroquinolones, but also clindamycin, daptomycin and linezolid in only 5.5 min. To our knowledge, to date, there are no published methods describing the same performance.

## 3. Materials and Methods

### 3.1. LC-MS Analysis

#### 3.1.1. Chemicals

Amoxicillin, ciprofloxacin, ofloxacin, clindamycin, cefotaxime, and piperacillin were purchased from Sigma (St. Gallen, Louis, MO, USA). Monohydrate cefepime dichlorhydrate was supplied from Gerda (Paris, France). Trihydrate meropenem and sodium cefoxitin were supplied from Panpharma (Fougères, France). Pentahydrate ceftazidime and sodium cefazolin were supplied from Mylan (Saint Priest, France). Ertapenem was supplied from MSD (Puteaux, France). Daptomycin was supplied from Medac SAS (Lyon, France). Aztreonam was supplied from Sanofi Aventis (Gentilly, France). Linezolid was supplied from Fresenius Kabi (Sevres, France). Internal standards (amoxicillin-D_4_, cefazolin-^13^C_2_^15^N, meropenem-D_6_, ofloxacin-D_8_, ciprofloxacin-D_8_, piperacillin-D_5_, cefotaxime-D_3_, and linezolid-D_3_) were purchased from TRC (Toronto, ON, Canada). Acetonitrile, methanol, formic acid, and water, all LC-MS hypergrade for mobile phase, were obtained from Biosolve (Dieuze, France). Plasma was purchased from the French Blood Bank (“Etablissement Français du Sang”, EFS, Reims, France).

#### 3.1.2. Chromatographic and Mass-Spectrometric Conditions

An ultra-performance liquid chromatographic system with an Ultimate 3000 high pressure pump (Thermo Fisher Scientific, San Jose, CA, USA) coupled with a triple quadrupole Quantis mass spectrometer (ThermoFisher Scientific, San Jose, CA, USA) was used for the development and validation of the method. Two microliters of the sample were injected. Chromatographic separation was obtained with a Waters Acquity HSS T3 1.8 μM (2.1 × 50 mm) UPLC column (Waters Corp; Milford, MA, USA), maintained at 35 °C. Mobile phases consisted of water + formic acid 0.1% (*v*/*v*) (MP-A) and acetonitrile + formic acid 0.1% (*v*/*v*) (MP-B). A programmed mobile-phase gradient was used at a flow rate of 0.3 mL/min (Table 5). The time of analysis and acquisition was 5.5 min, including re-equilibration.

Heated electro-spray ionisation in positive mode was performed with the following settings: sheat gas, 45 arbitrary units (AU); auxiliary gas, 7 AU; sweep gas, 2 arbitrary units set by the manufacturer (AU); spray voltage, static, 4 kV positive ion; ion transfer capillary temperature, 325 °C; and vaporiser temperature, 350 °C. Mass spectrometry was performed using parallel reaction monitoring mode (PRM). The settings for acquisition were as follows: Q1 resolution (FWHM), 0.7; Q3 resolution (FWHM), 1.2; CID gas (mTorr), 1.5. Dwell time and RF Lens were optimised for each compound. Energies of the collision were optimised for each transition (Table 1). A mass calibration check was performed every three months, and mass calibration was realised every six months in a positive and negative mode according to the manufacturer’s recommendations using an external calibration solution (ThermoScientific, San Jose, CA, USA). TraceFinder Forensic 4.1 was used for LC-MS, acquisition and processing.

#### 3.1.3. Preparation of Stock Solutions, Calibration Standards and Quality Control Samples

Stock solutions were prepared in water except for linezolid (prepared in DMSO) and ciprofloxacin, ciproflocaxin-D_8_, cefotaxime, cefotaxime-D_3_ and piperacillin (prepared in methanol). Stock solution concentrations were 1 g/L (amoxicillin, amoxicillin-D_4_, cefotaxime, ciprofloxacin, ciprofloxacin-D_8_, ofloxacin, ofloxacin-D_8_, and piperacillin), 2 g/L (cefazolin-^13^C_2_^15^N, cefotaxime-D_3_, linezolid, linezolid-D_3_, meropenem-D_6_, and piperacillin-D_5_), 10 g/L (clindamycin), 50 g/L (cefepime and meropenem), 100 g/L (daptomycin) and 200 g/L (aztreonam, ceftazidime, cefazolin, cefoxitin, and ertapenem). These solutions can be stored for six months at −80 °C, except for meropenem (two months at −80°C). In order to prepare the calibration standards (STD), the stocks solutions were diluted with an appropriate volume of methanol into a working solution (500 mg/L for aztreonam, 200 mg/L for amoxicillin, cefazolin, cefepime, cefotaxime, ceftazidime, daptomycin, ertapenem, and piperacillin, 100 mg/L for cefoxitin, and meropenem, 50 mg/L for linezolid, 10 mg/L for ciprofloxacin, clindamycin, and ofloxacin). This solution can be stored for 1 week at 4 °C. Calibration standards were prepared by diluting the working solution in blank plasma (half dilution at each calibration standards). The calibration range was designed with 6 calibration points including the zero. The calibration standard concentrations ranged from 15.625 to 500 mg/L for aztrenoam, 6.25 to 200 mg/L (amoxicillin, cefazolin, cefepime, cefotaxime, ceftazidime and piperacillin), 3.125 to 100 mg/L (cefoxitin, daptomycin, ertapenem and meropenem), 1.563 to 50 mg/L for linezolid, and 0.313 to 10 mg/L for ciprofloxacin, clindamycin, and ofloxacin. The concentrations of the calibration ranges were chosen and adjusted according to the concentration data for each antibiotic reported in the literature [7,29,33,35]. Internal standard working solution was prepared in methanol to obtain a final concentration of 60 mg/L for piperacillin-D_5_, 30 mg/L for amoxicillin-D_4_, cefotaxime-D_3_, cefazolin-^13^C_2_^15^N and meropenem-D_6_, 10mg/L for linezolid-D_3_ and 5 mg/L for ciprofloxacin-D_8_ and ofloxacin-D_8_. Quality controls at low, medium, and high levels (QCL, QCM and QCH) were prepared in our laboratory at different concentrations (Supplementary File: Table S5).

#### 3.1.4. Sample Processing

Twenty microliters of internal standard working solution were added to 100 µL of the plasma sample. A deproteinisation using 300 µL of methanol was performed. For calibration standards, 100 µL of working solution and 200 µL of methanol were added to 100 µL of blank plasma matrix. After vortex mixing for 60 s, the sample was centrifuged at 10,000× *g* for 5 min. The supernatant (50 µL) was recovered, and 200 μL of water (LC-MS hypergrade) containing 0.1% (*v*/*v*) formic acid was added.

### 3.2. Validation Procedure

For validation, antibiotics were divided into 2 pools (pool 1: amoxicillin, cefazolin, cefepime, cefoxitin, ceftazidime, ciprofloxacin, ertapenem, meropenem, and ofloxacin; pool 2: aztreonam, cefotaxime, clindamycin, daptomycin, linezolid, and piperacillin). Validation was conducted in accordance with international recommendations [39,40,44]. Linearity, precision, accuracy, selectivity, matrix effect, carryover, and stability assays were performed as previously described [21,41].

#### 3.2.1. Linearity

The limit of detection (LOD) was considered to be the lowest signal that the system could detect without confusing it with the noise. Therefore, the LOD was equivalent to the mean of 10 blank matrix concentrations plus 3 standard deviations or the value above the 95% confidence interval of the noise. The lower limit of quantitation (LLOQ) was considered to be the lowest concentration that the system was able to quantify with accuracy between 80% and 120% and an RSD value for precision of less than 20%. These parameters were estimated on 6 samples. The upper limit of quantitation (ULOQ) was considered to be the highest concentration that the system was able to quantify, with accuracy between 80% and 120% and an RSD value for precision of less than 20%. These parameters were estimated on 6 samples as well. Different weighting functions were tested for each analyte to select the regression calibration (linear. 1/X. 1/X^2^. 1/Y. quadratic…), as described previously [21]. To select the best regression model, we initially considered the bias of the quality controls and the model with the lowest bias on 3 levels of quality controls. The best R^2^ and the lowest absolute sum of square was also selected for each regression model. The extra-sum-of-squares F test, which is able to select the simplest model and a *p* value less than 0.2, was also performed. Finally, the residual distribution was observed for both the linear and quadratic regression models (y_observed_ − y_theorical_ as a function of different concentration levels) [21,39].

#### 3.2.2. Precision and Accuracy

Precision and accuracy were evaluated from the QC. Within-run and between-run accuracy and precision were assessed by analysing 15 samples per level. Precision was expressed as the relative standard deviation, which should not exceed ±15% for QC, and ±20% at LLOQ and ULOQ. For accuracy, the mean concentration should be within ±15% from the target concentration at each tested level, except for LLOQ and ULOQ (±20%). For accuracy, inter-laboratory assays were performed with QC samples.

#### 3.2.3. Selectivity

Ten plasma samples from donors were pre-treated and analysed individually as blanks to investigate interferences. As recommended, the absence of interfering components was accepted when the blank responses were lower than 20% of the LLOQ for the analytes and 5% for the corresponding internal standard.

#### 3.2.4. Matrix Effect

The matrix effect was evaluated according to the FDA (U.S. Food and Drug Administration), EMA (European Medecines Agency), and Matuszewsky et al. [39,40,44]. Six biological matrices of plasma from different sources were spiked after extraction with analytes (at 3-fold LLOQ and 80% of ULOQ) and with internal standards. The matrix effect factor was calculated by comparing the area under the peak derived from the matrix spiked after extraction and the area under the peak of a pure solution in the same concentration. The normalised factor of the matrix effect was determined for each matrix and analyte by comparing the matrix factor of the analyte and the matrix factor of the appropriate internal standard. The relative standard deviation of the normalised factors must be less than 20% [39,40,44].

#### 3.2.5. Stability

The stability was evaluated by 3 assays. The first test concerned the stability of the working solution used to carry out the calibration range. The stability of the standard solutions and IS solutions was evaluated by comparing a solution prepared for 1 week and stored at +4 °C with a freshly prepared solution. The second test was performed on the freeze-dried then reconstituted and frozen quality controls. The stability of the freeze-dried quality controls was assayed over a 3-month period at −20 °C. After reconstitution, stability of the quality controls was assayed for one week at −80 °C. Finally, the last test was performed on the extract to evaluate the post-preparative stability. Post-preparative stability was evaluated by keeping in an autosampler (+10 °C) the processed samples placed in glass vials for 3 h, 6 h, 12 h, and 24 h. All calibration standards as well as quality controls were used for this assay. The areas under the chromatographic peaks were compared as well as the areas normalised to the internal standard. For each analyte, a ratio of area under the curve (H0) to area under the curve (H24) normalised by the internal standards of 1.00 ± 15% and an RSD of less than 15% will ensure satisfactory post-preparative stability.

#### 3.2.6. Carry over Effects

Blank samples (*n* = 6) were analysed following the high concentration standard for the carry-over assay. As recommended, the signal should not be greater than 20% of the LLOQ and 5% of the IS [39,40].

### 3.3. Statistical Analysis

GraphPad Prism 5.0 (GraphPad Software, San Diego, CA, USA) was used for statistical analysis. Data are described as mean and standard deviation, except for Figure 3 of the matrix effect, which is described by the median and interquartiles. More is described in the supplementary files (Table S6).

## 4. Conclusions

The unstable status of patients in critical care units associated with intra- and inter-individual variability results in unpredictable antibiotic concentrations. Therapeutic drug monitoring of antibiotics represents a major asset in the management of infections maximising antibacterial efficacy and minimising toxicity.

In this work, we described the development and full validation of a precise, sensitive and accurate UPLC-MS/MS method that is able to simultaneously quantify 15 antibiotics, including beta-lactams, linezolid, fluoroquinolones, daptomycin, and clindamycin. The assay required small volumes of the biological sample and a simple pre-treatment. This method was easy to implement, and after validation, this new assay was successfully applied to routine analysis.

## Figures and Tables

**Table 1:** Retention time (RT), transition of precursors and fragments ions, mass spectrometry parameters for 15 antibiotics and their respective internal standards.

Compound Name	Retention Time (min)	Precursor	Precursor (*m*/*z*)	Products (*m*/*z*) Quantification and Confirmation	Collision Energy (eV)	Dwell Time (ms)	RF Lens (V)
Amoxicillin	2.44	[M + H]^+^	366.125	114.042; 349.125	20.00; 8.58	10	89
Aztreonam	2.61	[M + H]^+^	436.096	313.054; 356.125	14.48; 9.59	38	124
Cefazolin	3.05	[M + H]^+^	455.170	156.113; 323.042	15.28; 10.43	12	111
Cefepime	2.40	[M + H]^+^	241.150	84.155; 227.071	18.44; 10.39	10	75
Cefotaxime	2.82	[M + H]^+^	456.152	167.057; 324.125	19.49; 13.59	12	139
Cefoxitin	3.29	[M + NH_4_]^+^	445.170	339.054; 367.042	12.88; 8.71	20	102
Ceftazidime	2.56	[M + 2H]^2+^	274.150	80.125; 126,042	13.89; 22.90	8	96
Ciprofloxacin	2.81	[M + H]^+^	332.162	231.125; 314.125	35.96; 19.91	12	154
Clindamycin	3.02	[M + H]^+^	425.300	126.208; 377.280	28.63; 19.83	12	164
Daptomycin	3.58	[M + 2H]^2+^	811.000	159.000; 640.667	46.00; 20.00	25	204
Ertapenem	2.80	[M + H]^+^	476.150	346.208; 432.137	14.31; 8.71	12	117
Linezolid	3.20	[M + H]^+^	338.200	195.000; 296,083	22.00; 18.00	15	157
Meropenem	2.58	[M + H]^+^	384.200	141.155; 340.208	15.19; 10.31	8	111
Ofloxacin	2.78	[M + H]^+^	362.205	261.137; 318.137	26.10; 18.40	10	154
Piperacillin	3.52	[M + H]^+^	518.330	143.125; 160.125	19.66; 10.56	15	156
Amoxicillin-D_4_	2.44	[M + H]^+^	370.130	114.042; 353.130	20.00; 8.58	10	89
Cefazolin-^13^C_2_^15^N	3.05	[M + H]^+^	458.170	156.113; 326.042	15.28; 10.43	12	111
Cefotaxime-D_3_	2.82	[M + H]^+^	459.152	167.054; 327,125	19.49; 13.59	12	139
Ciprofloxacin-D_8_	2.81	[M + H]^+^	340.160	235.130; 322.130	35.96; 19.91	12	154
Linezolid-D_3_	3.20	[M + H]^+^	341.200	195.000; 296.800	22.00; 18.00	15	157
Meropenem-D_6_	2.58	[M + H]^+^	390.200	147.210; 346.208	15.19; 10.00	8	111
Ofloxacin-D_8_	2.78	[M + H]^+^	370.210	265.140; 326.140	26.10; 18.40	10	154
Piperacillin-D_5_	3.52	[M + H]^+^	523.330	143.125; 160.125	19.66; 10.56	15	156

**Table 2:** Limit of detection (LOD), lower limit of quantification (LLOQ), upper limit of quantification (ULOQ), and calibration curve parameters (*n* = 6). Results were expressed as relative standard deviation (%) for precision and biais (%) for accuracy.

Compound Name	Internal Standard	LOD (mg/L)	LLOQ (mg/L)	Precision of LLOQ (20%)	Accuracy of LLOQ (80–120%)	ULOQ (mg/L)	Precision of ULOQ (20%)	Accuracy of ULOQ (80–120%)	Calibration Curve (ax^2^ + bx + c)	r^2^
Amoxicillin	Amoxicillin-D_4_	0.1	1.6	1.9	98.3	200	1.2	102.0	−1.93 × 10^−4^x^2^ + 3.31 × 10^1^x + 0.27794	0.9990
Aztreonam	Piperacillin-D_5_	0.1	3.9	2.5	113.2	500	2.0	108.5	−4.70 × 10^−5^x^2^ + 0.27481x + 0.005967	0.9993
Cefazolin	Cefazolin-^13^C_2_^15^N	0.01	1.6	1.9	97.7	200	1.1	101.9	−1.84 × 10^−4^x^2^ + 0.1663x + 0.000542	0.9999
Cefepime	Cefazolin-^13^C_2_^15^N	0.01	1.6	1.6	95.8	200	2.1	100.9	−1.23 × 10^−4^x^2^ + 0.1077x + 0.000992	0.9997
Cefotaxime	Cefotaxime-D_3_	0.1	1.6	2.0	94.0	200	2.2	100.5	−2.29 × 10^−4^x^2^ + 0.21423x + 00.2344	0.9999
Cefoxitin	Cefazolin-^13^C_2_^15^N	0.01	0.6	1.3	118.39	100	1.2	100.6	−1.16 × 10^−4^x^2^ + 0.16816x + 0.000769	0.9998
Ceftazidime	Cefazolin-^13^C_2_^15^N	0.02	1.6	1.7	93.0	200	1.4	100.3	−7.75 × 10^−5^x^2^ + 0.08612x + 0.000919	0.9998
Ciprofloxacin	Ciprofloxacin-D_8_	0.001	0.1	3.5	99.8	10	1.2	97.8	2.15 × 10^−3^x^2^ + 1.5345x + 0.003918	0.9998
Clindamycin	Cefazolin-^13^C_2_^15^N	0.002	0.1	1.4	97.3	10	0.9	96.4	8.85 × 10^−3^x^2^ + 1.49766x + 0.000190	0.9998
Daptomycin	Cefazolin-^13^C_2_^15^N	0.3	1.6	1.6	109.9	200	1.5	112.5	−2.78 × 10^−5^x^2^ + 0.087183x + 0.002125	0.9996
Ertapenem	Meropenem-D_6_	0.004	0.8	2.7	102.6	100	1.2	100.9	2.72 × 10^−4^x^2^ + 0.32696x + 0.004432	0.9996
Linezolid	Linezolid-D_3_	0.004	0.4	1.6	87.8	50	0.6	98.5	8.92 × 10^−6^x^2^ + 0.33976x + 0.000520	0.9999
Meropenem	Meropenem-D_6_	0.003	0.8	3.6	100.0	100	1.5	102.7	6.52 × 10^−6^x^2^ + 0.19146x + 0.10970	0.9998
Ofloxacin	Ofloxacin-D_8_	0.006	0.1	2.6	100.7	10	0.9	99.7	−3.12 × 10^−3^x^2^ + 0.66686x + 0.000875	0.9998
Piperacillin	Piperacillin-D_5_	0.03	1.6	2.0	87.8	200	2.0	96.6	−9.61 × 10^−5^x^2^ + 0.4253x + 0.003480	0.9999

**Table 3:** Intra-assay and inter-assay for freeze-dried quality controls (3 levels: QC low, QC medium and QC high) (*n* = 10). Results were expressed as relative standard deviation (%) for precision and biais (%) for accuracy.

	QC Low				QC Medium				QC High			
	Intra-Assay		Inter-Assay		Intra-Assay		Inter-Assay		Intra-Assay		Inter-Assay	
	Precision	Accuracy	Precision	Accuracy	Precision	Accuracy	Precision	Accuracy	Precision	Accuracy	Precision	Accuracy
Amoxicillin	1.3	109.0	8.1	101.8	1.9	95.7	7.1	106.5	2.2	111.1	6.2	104.1
Aztreonam	1.8	100.1	10.2	90.3	1.9	101.2	9.2	90.0	2.4	103.3	5.7	108.5
Cefazolin	1.8	91.6	5.3	91.4	2.5	91.7	4.8	105.3	2.2	106.6	6.1	104.0
Cefepime	2.2	101.7	4.5	97.4	2.8	104.3	4.2	99.8	2.5	101.2	6.4	95.5
Cefotaxime	1.6	104.7	10.3	104.1	2.2	107.8	8.8	104.5	0.9	112.3	6.1	114.4
Cefoxitin	1.3	93.2	8.9	95.1	2.7	96.0	9.1	98.8	2.0	99.7	12.5	106.3
Ceftazidime	2.1	105.2	6.6	104.3	3.2	105.1	5.7	102.7	2.7	100.9	6.2	100.3
Ciprofloxacin	1.1	90.9	6.6	88.9	1.9	94.2	5.3	91.1	2.3	94.9	6.3	91.3
Clindamycin	1.6	110.0	6.5	89.4	2.3	109.0	5.3	91.8	1.2	112.7	6.1	97.0
Daptomycin	1.8	110.2	11.3	92.4	2.2	111.5	10.7	91.7	1.2	110.2	8.2	97.6
Ertapenem	1.5	90.7	8.4	86.3	2.6	103.8	7.1	94.7	1.5	113.0	8.1	100.8
Linezolid	2.1	87.9	6.1	89.3	2.2	90.9	5.2	90.5	1.1	91.6	3.1	95.3
Meropenem	1.9	111.8	8.1	103.3	2.7	112.1	6.6	107.5	2.1	112.4	8.8	107.9
Ofloxacin	2.1	110.6	7.6	92.7	2.3	113.4	4.9	89.6	1.8	104.1	5.1	85.9
Piperacillin	2.1	109.1	9.6	98.3	2.7	114.4	7.9	102.0	2.9	113.6	5.6	104.0

**Table 4:** Routine application of our method: therapeutic monitoring of 9 beta-lactams on patient plasma samples. Data were extracted over a three-month period. Cmax (peak plasma concentration), Cmin (minimum plasma concentration), Cont.inf (continuous infusion concentration).

	Timing	Samples (n)	Patients (n)	Mean Conc (Min–Max)	% of Concentrations Below the Reference Values	% of Concentrations Over the Reference Values
Amoxicillin	Cmin	92	58	52.8 (1.6–345.6)	51.1	17.4
Cefazolin	Cmin	10	8	66.3 (17.6–201.9)	22.2	22.2
Cefepime	Cmin	27	19	42.2 (1.6–158.7)	11.1	40.7
Cefotaxime	Cmin	23	5	63.6 (0.03–121.5)	13.1	47.8
Cefotaxime	Cont.inf	5	4	66.18 (25.3–140)	0	40
Ceftazidime	Cont.inf	33	24	71.3 (5.50–172.5)	28.1	40.6
Ertapenem	Cmin	4	2	16.4 (11.2–29.7)	0	100
Meropenem	Cmin	6	4	16.4 (0.8–40.9)	16.6	33.3
Piperacillin	Cmin	9	6	69.7 (14.7–144.1)	66.6	0

**Table 5:** Mobile phase gradient parameters.

Time	Flow (mL/Min)	MP-A% Water + Formic Acid 0.1% (*v*/*v*)	MP-B% ACN+ Formic Acid 0.1% (*v*/*v*)	Curve
0.000	0.3	100	0	5
3.600	0.3	14.5	85.5	5
3.601	0.3	5	95.0	5
4.10	0.3	5	95.0	5
4.110	0.3	100	0	5
5.500	0.3	100	0	5

## Data Availability

Data is contained within the article or supplementary material.

## Supplementary Materials

The following are available online at https://www.mdpi.com/article/10.3390/ph14121214/s1. Figure S1: Residual distribution (y_observed_ − y_theorical_ as function of different concentration levels) with linear or quadratic regression for each compound. Data are expressed as mean ± standard devaition (*n* = 6). Figure S2: Focus on overlay (LLOQ and matrix blank): example of ertapenem and clindamycin. Table S1: Bias on 3 levels of quality controls for two regression model (linear and quadratic regression, *n* = 6). Table S2: Goodness-of-fit analysis. R^2^ and Absolute Sum of square were calculated for two models of regression of the calibration curve (linear and quadratic regression, *n* = 6). Table S3: Matrix effect for in 6 different blanks plasma samples at two concentrations for each compound (3-fold- LLOQ and 80% of ULOQ). RSD: relative standard deviation. Table S4: Stability assays: Evolution over 5 days of the working solutions kept at +4 °C and post-preparative stability results. Data are expressed as mean and RSD. Table S5: Target concentrations of quality control. Table S6: Mathematical equation for the accuracy and relative standard deviation.
