# Supplementary material for: Quantification of 15 Antibiotics Widely Used in the Critical Care Unit with a LC-MS/MS System: An Easy Method to Perform a Daily Therapeutic Drug Monitoring
Source: Pharmaceuticals (Basel). 2021 Nov 24;14(12):1214. doi: 10.3390/ph14121214 (PMC8703964; doi:10.3390/ph14121214)
Supplement: Supplementary file 1 [file pharmaceuticals-14-01214-s001.zip › pharmaceuticals-1421763-supplementary.pdf]

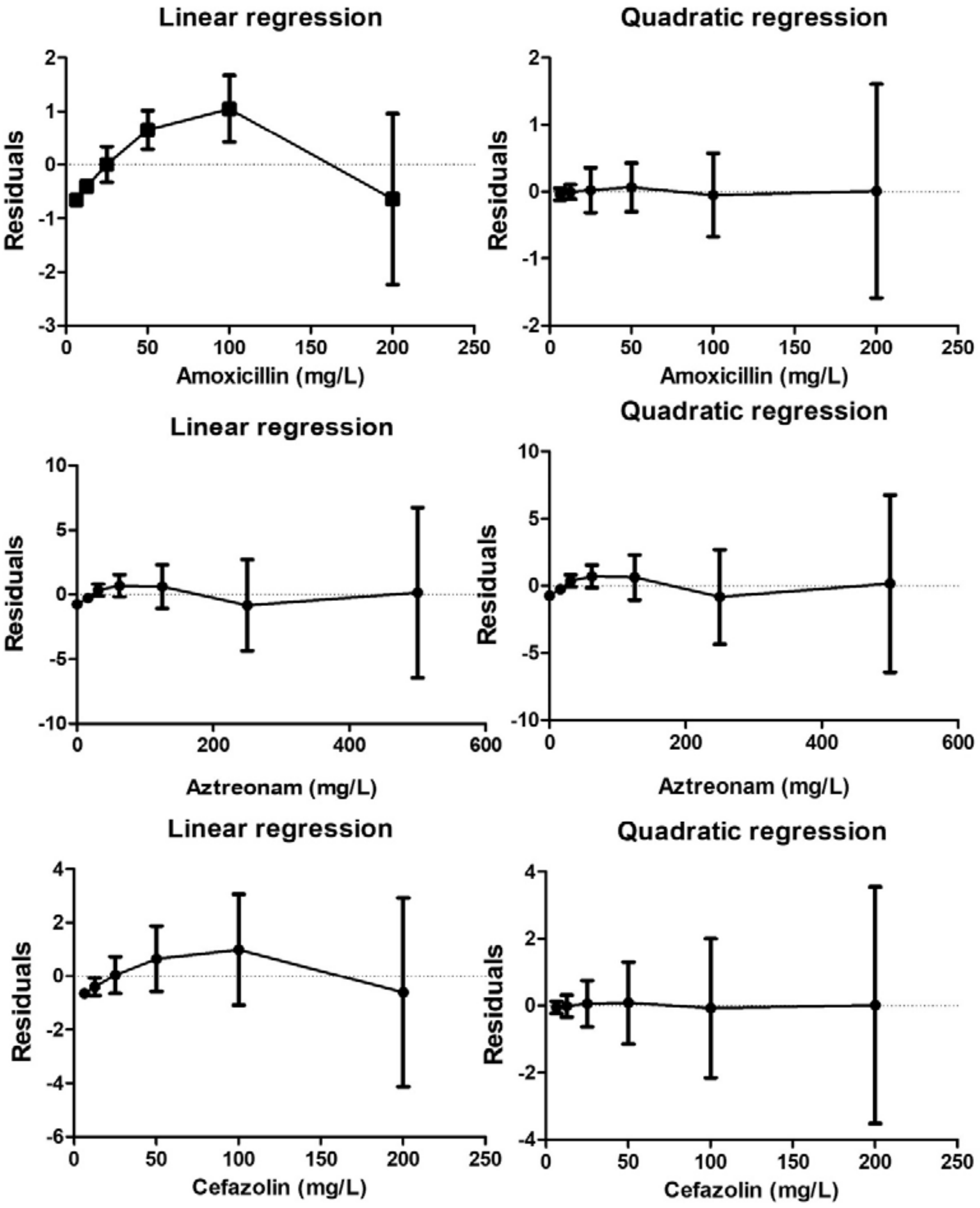
Linear regression
Quadratic regression
Residuals
Amoxicillin (mg/L)
Linear regression
Quadratic regression
Residuals
Aztreonam (mg/L)
Linear regression
Quadratic regression
Residuals
Cefazolin (mg/L)

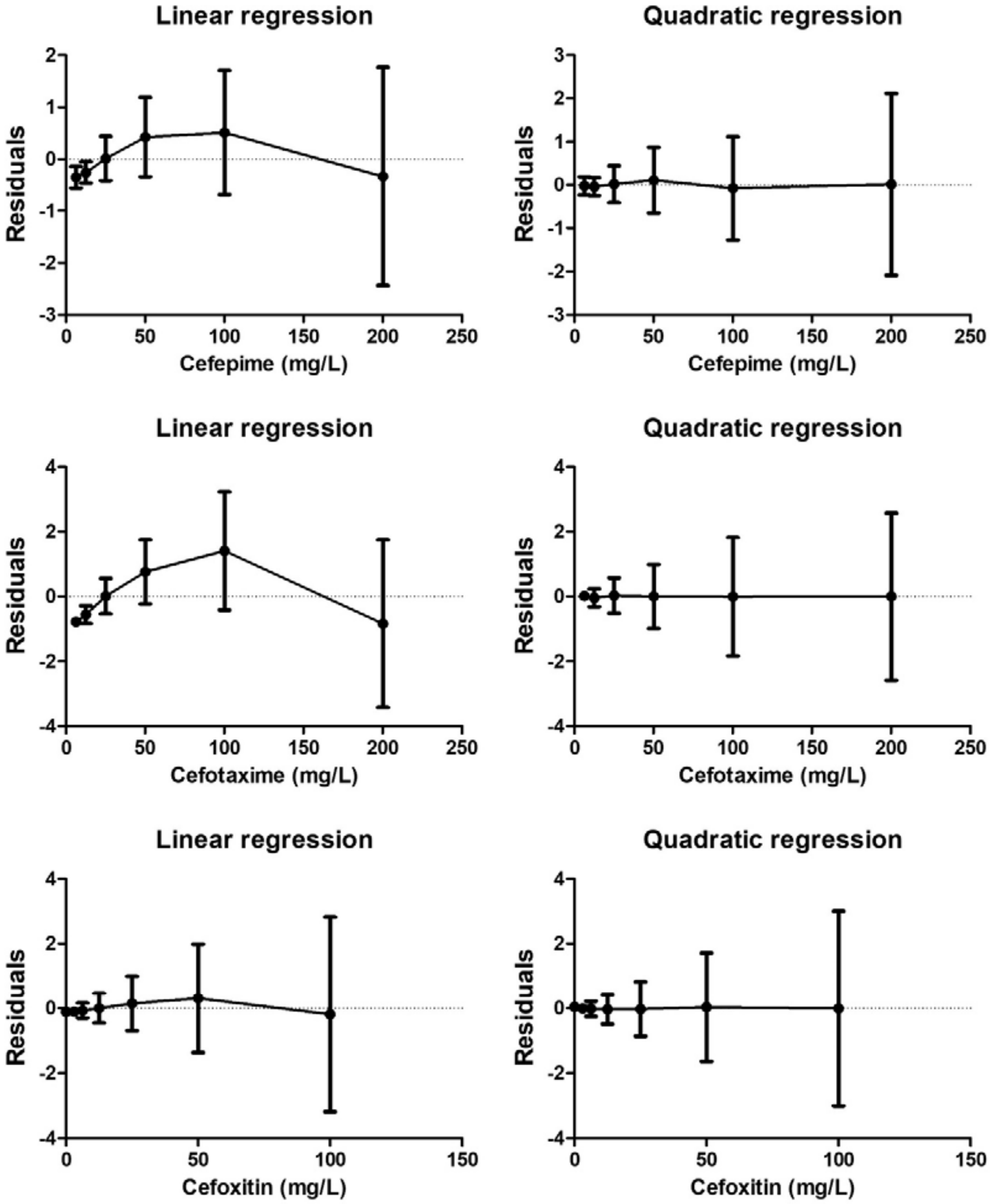

Linear regression
Quadratic regression
Residuals
Cefepime (mg/L)
Linear regression
Quadratic regression
Residuals
Cefotaxime (mg/L)
Linear regression
Quadratic regression
Residuals
Cefoxitin (mg/L)

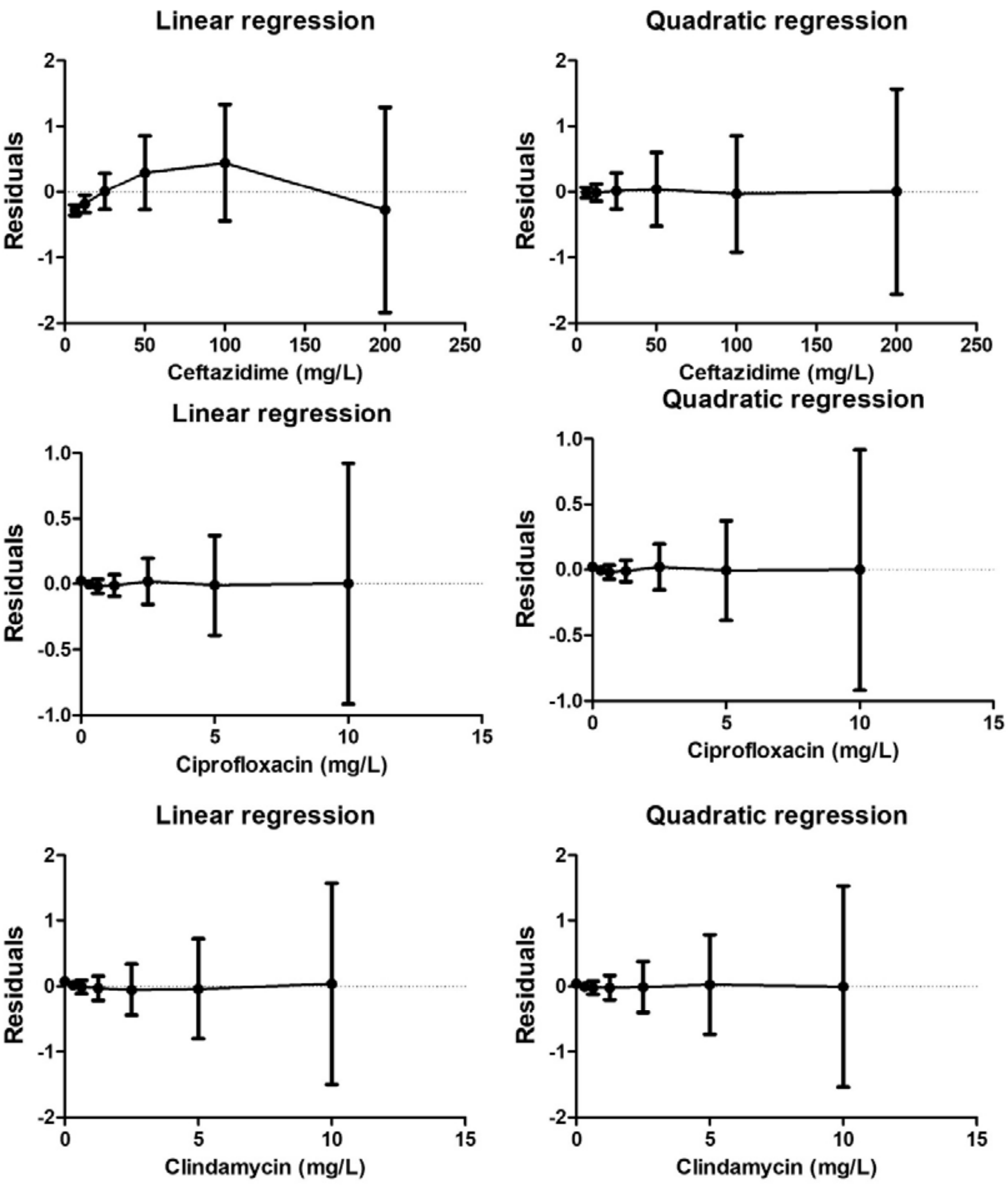
Linear regression
Quadratic regression
Residuals
Ceftazidime (mg/L)
Linear regression
Quadratic regression
Residuals
Ciprofloxacin (mg/L)
Linear regression
Quadratic regression
Residuals
Clindamycin (mg/L)

Figure S1 : Residual distribution (($y_{observed}$- $y_{theorical}$ as function of different concentration level) with linear or quadratic regression for each compound. Data are expressed as mean ± standard deviation (n=6)

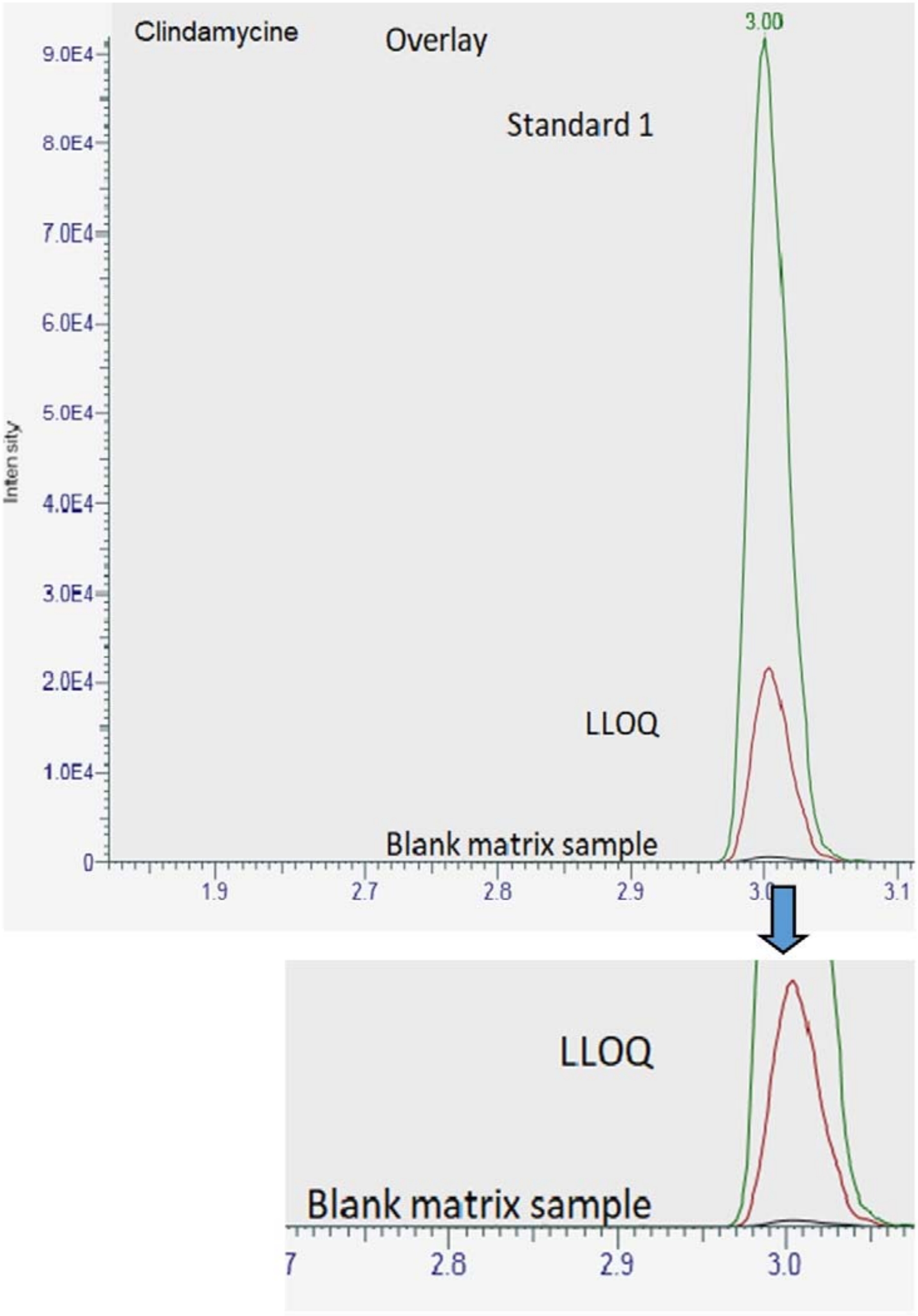

Figure S2: Focus on overlay (LLOQ and matrix blank): example of ertapenem and clindamycin

Table S1: Bias on 3 levels of quality controls for two regression models (linear and quadratic regression, n= 6).

	QC low bias (%)		QC medium bias (%)		QC high bias (%)	
	**Linear regression**	**Quadratic regression**	**Linear regression**	**Quadratic regression**	**Linear regression**	**Quadratic regression**
Amoxicillin	11.61	4.75	13.23	8.57	9.07	9.26
Aztreonam	-10.55	-10.17	-12.04	-9.46	-17.59	-15.06
Cefazolin	19.98	3.66	5.36	2.28	11.79	11.66
Cefepime	22.81	11.26	31.93	19.33	18.19	14.19
Cefotaxime	22.61	3.38	11.88	0.40	3.28	0.11
Cefoxitin	9.99	-6.37	6.58	-4.30	-2.73	-1.74
Ceftazidime	16.34	5.28	14.59	4.89	10.73	10.66
Ciprofloxacin	-5.17	-3.62	2.01	1.14	5.68	6.24
Clindamycin	-3.35	-3.37	-10.46	-8.31	-1.50	-1.23
Daptomycin	-10.73	-8.28	-12.78	-10.21	-13.91	-12.81
Ertapenem	8.45	6.54	13.37	11.71	8.44	8.23
Linezolid	6.52	3.54	4.62	2.83	6.21	5.01
Meropenem	15.85	10.43	15.53	11.86	14.66	10.02
Ofloxacin	-10.08	-9.62	-14.61	-10.61	0.80	0.74
Piperacillin	-11.45	-7.90	-9.66	-7.35	-9.78	-9.38

Table S2: Goodness-of-fit analysis. R^2 and Absolute Sum of square were calculated for two models of regression of the calibration curve (linear and quadratic regression, n =6).

	Linear regression		Quadratic regression	
	**R^2**	**Absolute Sum of square**	**R^2**	**Absolute Sum of square**
Amoxicillin	0.9977	42.58	0.9988	22.46
Aztreonam	0.9956	380.50	0.9957	375.8
Cefazolin	0.9625	151.10	0.9671	132.5
Cefepime	0.9700	52.92	0.9733	47.13
Cefotaxime	0.9819	97.10	0.9873	67.99
Cefoxitin	0.9571	77.99	0.9578	76.78
Ceftazidime	0.9755	29.32	0.9786	25.57
Ciprofloxacin	0.9954	7.20	0.9954	7.20
Clindamycin	0.9861	18.97	0.9862	18.90
Daptomycin	0.9299	85.52	0.9307	84.53
Ertapenem	0.9676	276.70	0.9682	271.6
Linezolid	0.9976	3.73	0.9976	3.73
Meropenem	0.9649	88.46	0.9649	88.42
Ofloxacin	0.9942	1.44	0.9945	1.37

Piperacillin	0.9935	202.3	0.9936	200.5

Table S3: Matrix effect in 6 different blank plasma samples at two concentrations for each compound (3-fold- LLOQ and 80 % of ULOQ). RSD: relative standard deviation

	3-fold- LLOQ				80 % ULOQ			
	Matrix factor		IS normalised matrix factor		Matrix factor		IS normalised matrix factor	
	Mean (AU)	RSD (%)	Mean (AU)	RSD (%)	Mean (AU)	RSD (%)	Mean (AU)	RSD (%)
Amoxicillin	0.72	11.9	0.88	9.7	0.92	2.6	1.0	2.7
Aztreonam	0.95	3.8	0.99	3.1	0.93	1.4	0.93	2.2
Cefazolin	0.73	14.8	0.86	11.3	0.92	2.5	1.04	1.0
Cefepime	0.74	14.4	0.88	10.5	0.91	3.2	0.99	0.9
Cefotaxime	0.95	3.7	0.99	1.3	0.91	1.7	0.98	2.1
Cefoxitin	0.72	10.1	0.85	6.8	0.83	5.67	0.95	5.35
Ceftazidime	0.76	14.7	0.85	11.8	0.92	2.21	1.05	2.60
Ciprofloxacin	1.14	5.7	1.04	4.5	0.98	1.51	1.07	2.15
Clindamycin	0.96	4.6	1.02	2.8	0.92	0.6	0.98	1.7
Daptomycin	0.90	4.0	0.96	3.1	0.88	1.0	0.94	2.6
Ertapenem	0.75	11.4	0.86	10.8	0.93	3.76	1.03	2.8
Linezolid	0.81	10.8	1.0	2.4	0.84	6.12	0.97	2.7
Meropenem	0.72	11.8	0.82	10.1	0.93	2.46	1.03	2.2
Ofloxacin	0.95	14.2	1.11	11.1	0.96	1.63	1.09	3.44
Piperacillin	1.01	5.5	1.05	3.4	0.96	1.7	0.98	3.4

Table S4: Stability assays : Evolution over 5 days of the working solutions kept at +4°C and post-preparative stability results. Data are expressed as mean and RSD.

Stability of Standard solutions and IS solutions

Compounds	Mean of Areas under the curve of standard 6 (n=5, day 1 to day 5)	RSD (%)
Amoxicillin	27451628	6.6
Aztreonam	23458505	5.8
Cefazolin	13842996	7.1
Cefepime	9133927	7.5
Cefotaxime	6824045	6.1
Cefoxitin	9394058	11.2
Ceftazidime	7469856	8.8
Ciprofloxacin	4924375	10.3
Clindamycin	5990753	6.4
Daptomycin	5933826	3.9
Ertapenem	14321930	11.3
Linezolid	12285472	7.0
Meropenem	7858607	8.7
Ofloxacin	7980404	8.3
Piperacillin	15262691	7.1
Amoxicillin-D_4	472857	6.4
Cefazolin-$^{13}C_2{}^{15}N$	545067	10.0
Cefotaxime-D_3	197816	9.3
Ciprofloxacin-D_8	470039	11.2
Meropenem-D_6	407526	14.4
Linezolid-D_3	800445	7.5
Meropenem-D_6	954139	9.3
Ofloxacin-D_8	192074	7.0
Piperacillin-D_5	27451628	6.6

Post-preparative stability

Compounds	Mean ratios of the area under the curve (H0) to area under the curve (H24) normalized by the internal standards (n=6)	RSD (%)
Amoxicillin	0.98	1.5
Aztreonam	0.92	1.9
Cefazolin	1.00	2.3
Cefepime	0.92	3.8
Cefotaxime	0.97	3.0
Cefoxitin	1.05	2.1
Ceftazidime	0.96	2.8
Ciprofloxacin	0.99	4.3
Clindamycin	0.93	3.4
Daptomycin	1.0	4.8
Ertapenem	1.55 (H24) – 1.01 (H3)	10.5 (H24) – 2.8 (H3)
Linezolid	0.97	1.0
Meropenem	1.00	5.2
Ofloxacin	0.98	1.8
Piperacillin	0.97	2.1

Table S5: Target concentrations of quality control

	Target QC low (mg/L)	Target QC medium (mg/L)	Target QC high (mg/L)
Amoxicillin	31.06	62.77	126.51
Aztreonam	88.21	176.94	369.77
Cefazolin	37.91	76.89	152.11
Cefepime	26.47	51.38	109.51
Cefotaxime	37.33	75.59	155.85
Cefoxitin	17.05	35.59	71.93
Ceftazidime	32.91	68.17	136.58
Ciprofloxacin	1.69	3.40	6.88
Clindamycin	1.78	3.73	7.34
Daptomycin	20.79	42.45	85.57
Ertapenem	17.10	33.75	65.84
Linezolid	9.05	18.39	37.67
Meropenem	14.98	30.35	71.01
Ofloxacin	1.79	3.60	7.26
Piperacillin	37.21	77.19	161.91

Table S6 : Mathematical equation for the accuracy and relative standard deviation

Accuracy	$\frac{\text{(measured concentration - target concentration)}}{\text{target concentration}} \times 100$
Relative standard deviation	$\frac{\text{standard deviation}}{\text{mean}} \times 100$
